# A New Underwater Acoustic Signal Denoising Technique Based on CEEMDAN, Mutual Information, Permutation Entropy, and Wavelet Threshold Denoising

**DOI:** 10.3390/e20080563

**Published:** 2018-07-28

**Authors:** Yuxing Li, Yaan Li, Xiao Chen, Jing Yu, Hong Yang, Long Wang

**Affiliations:** 1 School of Marine Science and Technology, Northwestern Polytechnical University, Xi’an 710072, China; 2 School of Electronic and Engineering, Xi’an University of Posts and Telecommunications, Xi’an 710121, China

**Keywords:** denoising, CEEMDAN, mutual information, permutation entropy, wavelet threshold denoising, chaotic signal, underwater acoustic signal

## Abstract

Owing to the complexity of the ocean background noise, underwater acoustic signal denoising is one of the hotspot problems in the field of underwater acoustic signal processing. In this paper, we propose a new technique for underwater acoustic signal denoising based on complete ensemble empirical mode decomposition with adaptive noise (CEEMDAN), mutual information (MI), permutation entropy (PE), and wavelet threshold denoising. CEEMDAN is an improved algorithm of empirical mode decomposition (EMD) and ensemble EMD (EEMD). First, CEEMDAN is employed to decompose noisy signals into many intrinsic mode functions (IMFs). IMFs can be divided into three parts: noise IMFs, noise-dominant IMFs, and real IMFs. Then, the noise IMFs can be identified on the basis of MIs of adjacent IMFs; the other two parts of IMFs can be distinguished based on the values of PE. Finally, noise IMFs were removed, and wavelet threshold denoising is applied to noise-dominant IMFs; we can obtain the final denoised signal by combining real IMFs and denoised noise-dominant IMFs. Simulation experiments were conducted by using simulated data, chaotic signals, and real underwater acoustic signals; the proposed denoising technique performs better than other existing denoising techniques, which is beneficial to the feature extraction of underwater acoustic signal.

## 1. Introduction

With the development of ocean scientific technology, the use and protection of the oceans have attracted more extensive attention. Because of the complexity of the marine environment and the time-varying nature of the underwater acoustic channel, it is more difficult to detect and reduce the noise of underwater acoustic signals [1,2]. Therefore, the research on underwater acoustic signal processing method and its application are very essential and important in the field of underwater acoustic. Underwater acoustic signals not only are nonlinear, non-stationary, and non-Gaussian, but also chaos and fractal, traditional signal processing methods based on the classic Fourier analysis are not suitable for underwater acoustic signals such as short-time Fourier transform, Fourier transform, Wigner–Ville, and wavelet transform [3,4]. Therefore, finding a suitable method is the key to analysis underwater acoustic signal.

EMD and its improved algorithms are suitable for analyzing nonlinear, non-stationary, and non-Gaussian signals. Furthermore, EMD and its improved algorithms are self-adaptive and based on characteristic time scale of the data itself. However, EMD has the phenomena of mode mixing due to intermittency, which hinders the development and application of EMD [5]. In order to reduce its influence, many improved algorithms are presented such as EEMD, complementary EEMD (CEEMD), and complete EEMD with adaptive noise (CEEMDAN) [6,7,8]. The proposed method follows a study of the statistical characteristics of white noise, involves a noise-assisted analysis, and adds white noise of a uniform frequency distribution into EMD to avoid mode mixing. EEMD is a noise-assisted analysis algorithm to avoid mode mixing by adding white noise. However, this improved algorithm raises two new problems, one is the difference in IMF numbers and the other is the introduction of extra noise. CEEMD can avoid adding extra noise using positive and negative white noises, however, it still cannot make the number of IMF consistent by each decomposition. CEEMDAN can solve this problem because it only decomposes the first IMF for each decomposition; it has better decomposition effect and lower computational cost than EEMD and CEEMD.

EMD and its improved algorithms are widely used in different fields [9,10,11]. In the field of fault diagnosis, a fault detection and diagnosis algorithm is proposed based on EEMD and the particle swarm optimization algorithm previous reported [12]. In a previous paper [13], a new health degradation monitoring and early fault diagnosis for rolling bearing signal is proposed using CEEMDAN and improved multi-scale entropy. In another past paper [14], a fault diagnosis algorithm for planetary gear is put forward based on CEEMDAN, PE, and an adaptive neuro-fuzzy inference system. In the field of medicine, CEEMDAN is carried out toanalyze heart rate variability in electrocardiogram (ECG) signals [15]. In addition, the EMD algorithm was used for analyzing focal electroencephalogram (EEG) signals and 3D EEG signals [16,17]. In the underwater acoustic area, EMD and EEMD algorithms are carried out for extracting the characteristics of underwater acoustic signals [1,18]. In conclusion, a large number of studies have proved the effectiveness and feasibility of the EMD and its improved algorithms.

In recent years, many denoising methods based on EMD and its improved algorithms have been proposed [19,20]. In a previous paper [21], a denoising algorithm for gear signals is proposed based on CEEMDAN, PE and peak filtering, the IMF spectra are obtained by CEEMDAN, and the PEs of IMFs are calculated to identify whether the IMFs require denoising by peak filtering, the filtered and the others IMFs are reconstructed finally. In a previous paper [22], a ECG signal denoising algorithm is put forward using CEEMDAN and wavelet threshold denoising. In addition, denoising algorithms for the impact signal and friction signal are proposed using CEEMDAN combined with fuzzy entropy and MI in past papers [23,24]. However, there has been no previous studies on underwater acoustic signal denoising based on CEEMDAN as far as we know [25,26]. Moreover, among the existing denoising algorithms, IMFs are usually divided into two parts: noise IMFs and real IMFs, and there are some limitations in this division.

In this paper, a hybrid denoising algorithm for underwater acoustic signals is presented by taking advantage of CEEMDAN, MI, PE, and wavelet threshold denoising. Compared with the existing denoising algorithms, the proposed algorithm divides IMFs into three parts, which is beneficial to signal denoising. This paper is organized as follows: Section 2 is the basic methods of CEEMDAN, MI, PE, and wavelet threshold denoising; in Section 3, the underwater acoustic signal denoising technique is presented; in Section 4, Section 5 and Section 6, the proposed denoising algorithm is applied to simulated data, chaotic signals, and real underwater acoustic signals respectively; finally, Section 7 is the conclusion.

## 2. Methods

### 2.1. CEEMDAN

**Figure 1:**
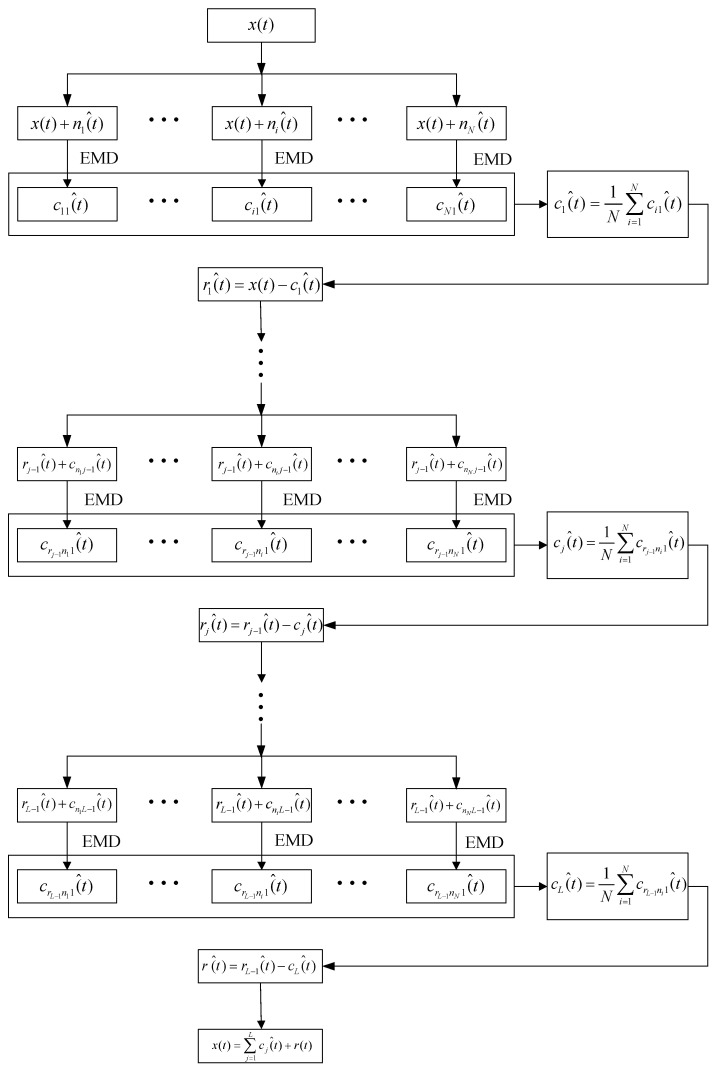
The flow chart of complete ensemble empirical mode decomposition with adaptive noise (CEEMDAN).

CEEMDAN, as an improved algorithm of EMD and EEMD, can adaptively decompose complex signal into IMFs in order. The specific steps of CEEMDAN are summarized as follows [8]:

(1) Add white noise $n_{i}\hat{(}t)$ to the target signal x(t):
  (1) $$x_{i}\hat{(}t)=x(t)+n_{i}\hat{(}t),\begin{array}{cc} & i=1,\;2,\;\cdots ,N \end{array}$$
(2) Decompose $x_{i}\hat{(}t)$ by EMD to obtain the first IMF $c_{i1}\hat{(}t)$ and residual mode $r_{i}(t)$:
  (2) $$\left(\begin{array}{c} x_{1}\hat{(}t)\\ x_{2}\hat{(}t)\\ \cdots \\ x_{i}\hat{(}t)\\ \cdots \\ x_{N}\hat{(}t) \end{array}\right)\overset{\mathrm{EMD}}{\to }\left(\begin{array}{cc} c_{11}\hat{(}t) & r_{1}(t)\\ c_{21}\hat{(}t) & r_{2}(t)\\ \cdots & \cdots \\ c_{i1}\hat{(}t) & r_{i}(t)\\ \cdots & \cdots \\ c_{N1}\hat{(}t) & r_{N}(t) \end{array}\right)$$
(3) Obtain the first IMF of CEEMDAN by calculating the mean of $c_{i}{}_{1}\hat{(}t)$:
  (3) $$c_{1}\hat{(}t)=\frac{1}{N}\sum_{i=1}^{N}c_{i}{}_{1}\hat{(}t)$$
(4) Obtain the residual mode of $c_{1}\hat{(}t)$:
  (4) $$r_{1}\hat{(}t)=x(t)-c_{1}\hat{(}t)$$
(5) Decompose white noise $n_{i}\hat{(}t)$ by EMD:
  (5) $$\left(\begin{array}{c} n_{1}\hat{(}t)\\ n_{2}\hat{(}t)\\ \cdots \\ n_{i}\hat{(}t)\\ \cdots \\ n_{N}\hat{(}t) \end{array}\right)\overset{\mathrm{EMD}}{\to }\left(\begin{array}{ccccc} c_{n_{1}1}\hat{(}t) & c_{n_{1}2}\hat{(}t) & \cdots & c_{n_{1}j}\hat{(}t) & r_{n_{1}}\hat{(}t)\\ c_{n_{2}1}\hat{(}t) & c_{n_{2}2}\hat{(}t) & \cdots & c_{n_{2}j}\hat{(}t) & r_{n_{2}}\hat{(}t)\\ \cdots & \cdots & \cdots & \cdots & \cdots \\ c_{n_{i}1}\hat{(}t) & c_{n_{i}2}\hat{(}t) & \cdots & c_{n_{i}j}\hat{(}t) & r_{n_{i}}\hat{(}t)\\ \cdots & \cdots & \cdots & \cdots & \cdots \\ c_{n_{N}1}\hat{(}t) & c_{n_{N}2}\hat{(}t) & \cdots & c_{n_{N}j}\hat{(}t) & r_{n_{N}}\hat{(}t) \end{array}\right)$$
  where $c_{n_{i}j}\hat{(}t)$ represents the j-th IMF of the i-th white noise, $r_{n_{i}}\hat{(}t)$ represents the residual mode of the i-th white noise. $E_{j}(s_{i}(t))$ is defined as a set which includes the j-th IMF of $s_{i}(t)$, $E_{1}(n_{i}\hat{(}t))$ is expressed as:
  (6) $$E_{1}(n_{i}\hat{(}t))={\left(\begin{array}{cccccc} c_{n_{1}1}\hat{(}t) & c_{n_{2}1}\hat{(}t) & \cdots & c_{n_{i}1}\hat{(}t) & \cdots & c_{n_{N}}\hat{(}t) \end{array}\right)}^{T}$$
(6) Construct signal $xnew_{1}(t)$ and decompose it by EMD (only decompose the first IMF):
  (7) $$xnew_{1}(t)=r_{1}\hat{(}t)+E_{1}(n_{i}\hat{(}t))$$
  (8) $$xnew_{1}(t)=r_{1}\hat{(}t)+\left(\begin{array}{c} c_{n_{1}1}\hat{(}t)\\ c_{n_{2}1}\hat{(}t)\\ \cdots \\ c_{n_{i}1}\hat{(}t)\\ \cdots \\ c_{n_{N}1}\hat{(}t) \end{array}\right)\overset{\mathrm{EMD}}{\to }\left(\begin{array}{c} c_{r_{1}n_{1}1}\hat{(}t)\\ c_{r_{1}n_{2}1}\hat{(}t)\\ \cdots \\ c_{r_{1}n_{i}1}\hat{(}t)\\ \cdots \\ c_{r_{1}n_{N}1}\hat{(}t) \end{array}\right)$$
(7) Obtain the second IMF $c_{2}\hat{(}t)$ and residual mode $r_{2}\hat{(}t)$ of CEEMDAN:
  (9) $$c_{2}\hat{(}t)=\frac{1}{N}\sum_{i=1}^{N}c_{r_{1}n_{i}}{}_{1}\hat{(}t)$$
  (10) $$r_{2}\hat{(}t)=r_{1}\hat{(}t)-c_{2}\hat{(}t)$$
(8) Obtain $xnew_{j-1}(t)$ and repeat step (6) and (7), $c_{j}\hat{(}t)$ and $r_{j}\hat{(}t)$ are expressed as:
  (11) $$xnew_{j-1}(t)=r_{j-1}\hat{(}t)+E_{j-1}(n_{i}\hat{(}t))$$
  (12) $$c_{j}\hat{(}t)=\frac{1}{N}\sum_{i=1}^{N}c_{r_{j-1}n_{i}}{}_{1}\hat{(}t)$$
  (13) $$r_{j}\hat{(}t)=r_{j-1}\hat{(}t)-c_{j}\hat{(}t)$$
(9) x(t) is expressed as:
  (14) $$x(t)=\sum_{j=1}^{L}c_{j}\hat{(}t)+r(t)$$
  where L represents the number of IMF by CEEMDAN, r(t) represents the residual mode. The flow chart of CEEMDAN is designed in Figure 1.

In this paper, we chose the CEEMDAN algorithm for the following reasons:

(1) CEEMDAN has better decomposition effect and lower computational cost than EEMD and CEEMD.
(2) CEEMDAN is suitable for analyzing non-linear, non-stationary and non-Gaussian signals, in theory, it can decompose all signals.
(3) CEEMDAN is self-adaptive and based on characteristic time scale of the data itself without basis function.

### 2.2. MI

For two discrete random variables X and Y, the MI can be defined as [27]:

(15) $$I(X;Y)=\sum_{y\in Y}\sum_{x\in X}p(x,y)\log(\frac{p(x,y)}{p(x)p(y)})$$

where p(x,y) is the joint probability distribution function of x and y, p(x) and p(y) are the marginal probability distribution functions of x and y, respectively.

The MI of continuous random variables can be expressed as a double integral:

(16) $$I(X;Y)=\int_{Y}\int_{X}p(x,y)\log(\frac{P(x,y)}{p(x)p(y)})dxdy$$

In probability theory and information theory, the mutual information of two random variables represents a measure of the interdependence of variables. If X and Y are independent, I(X;Y)=0. In addition, the symmetry of MI can be expressed as:

(17) I(X;Y)=I(Y;X)

Furthermore, the MI can also be expressed as:

(18) $$\begin{array}{ll} I(X;Y) & =H(X)-H(X|Y)\\ & =H(Y)-H(Y|X)\\ & =H(X)+H(Y)-H(X,Y)\\ & =H(X,Y)-H(X|Y)-H(Y|X) \end{array}$$

where H(X) and H(Y) are informationentropy, H(X|Y) and H(Y|X) are conditional entropy, H(X,Y) is joint entropy of X and Y.

**Figure 2:**
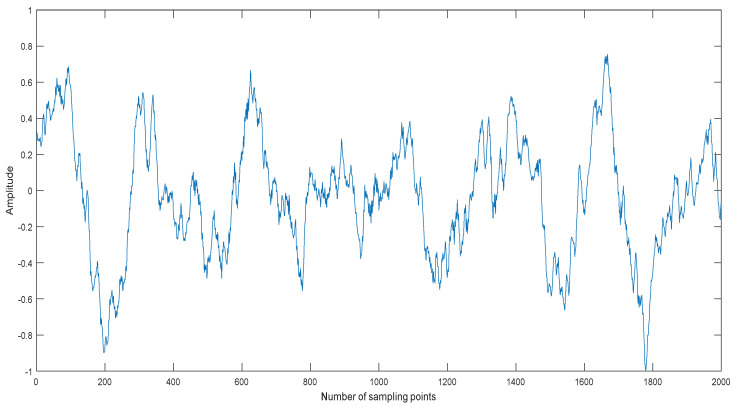
The normalized ship signal.

**Figure 3:**
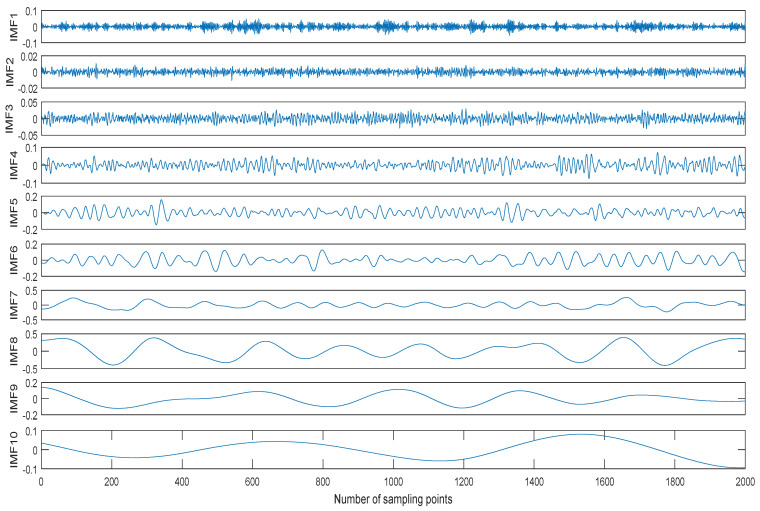
The decomposition result of the ship signal by CEEMDAN.

Usually the MIs between noise IMFs are different from ones between non-noise IMFs. We take a ship signal as an example. The normalized ship signal is shown as shown in Figure 2, the sampling frequency and the number of sampling points are 44.1 kHz and 2000, respectively. The decomposition result of the ship signal by CEEMDAN is shown in Figure 3. The center frequency distribution of IMFs and the MIs of two neighboring IMFs are shown in Table 1 and Table 2, where MI*_i_* represents the MI of IMF*_i_* and IMF*_i_*_+1_. As shown in Table 1 and Table 2, the center frequency decreases with the increase of IMF, the first three MI of IMFs are obviously less than the other ones of IMFs. According to the prior information of ship signal, its main frequency range is less than 5000Hz, the first three IMFs are noise IMFs, which is consistent with the judgment of MI. Therefore, we can use MI to identify noise IMFs in this paper, when the MI of IMF*_i_* and IMF*_i_*_+1_ increases, obviously more than the former MIs, the former *i* − 1 IMFs are considered as noise IMFs.

### 2.3. PE

PE is proposed by Bandt in [24]. The brief process of PE is as follows [28]:

(1) Reconstruct time series $X=\{x_{1},x_{2},\cdots ,x_{N}\}$:
  (19) $$\left\{ \begin{array}{l} \{x(1),x(1+\tau ),\cdots ,x(1+(m-1)\tau )\}\\ \begin{array}{ccc} & \vdots & \end{array}\\ \{x(j),x(j+\tau ),\cdots ,x(j+(m-1)\tau )\}\\ \begin{array}{ccc} & \vdots & \end{array}\\ \{x(K),x(K+\tau ),\cdots ,x(K+(m-1)\tau )\}\;(K=n-(m-1)\tau ) \end{array}\right.$$
  where τ and m are the time lag and embedding dimension.
(2) Rearrange each row vectorin ascending order:
  (20) x(i+(j1−1)τ)≤x(i+(j2−1)τ)≤⋯≤x(i+(jm−1)τ)
(3) Obtain a symbol-sequence for each row vector as:
  (21) S(g)=(j1,j2,⋯,jm) (g=1,2,⋯,l and l≤m!)
(4) Define PE as:
  (22) $$H_{P}(m)=-\sum_{j=1}^{l}P_{j}\ln P_{j}$$
  where $P_{j}$ is the probability of one symbol-sequence.
(5) Define normalized PE as:
  (23) $$H_{P}=H_{P}(m)/\ln(m!)$$

More detail about PE was described previously [29]. In this study, we set m=3 and τ=1 according to the suggestiondescribed previously [30]. In a previous paper [21], PE is used to identify noisy IMF. Therefore, in this paper, we choose PE to identifynoise-dominant IMF.

### 2.4. Wavelet Threshold Denoising

Signal denoising is an important research direction of signal processing. The wavelet transform has multi-resolution characteristics. One-dimensional noisy time series can be expressed as follows [31]:

(24) s(k)=f(k)+e(k),k=0,1,2,⋯,n

where f(k) is original signal, e(k) isnoise signal, s(k) is noisy signal.

Assuming that e(k) is Gaussian white noise, f(k) is usually represented as a low-frequency signal in practical engineering applications. Therefore, we can use the following methods to reduce noise. The specific steps are as follows:

(1) A proper wavelet basis function and decomposition level are selected to perform wavelet decomposition on the noisy signal.
(2) Threshold is performed by selecting an appropriate threshold method for high frequency coefficients at different decomposition scales.
(3) The low frequency coefficient of wavelet decomposition and the thresholdhigh frequency coefficient of different scales are used to reconstruct.

Wavelet thresholding with different thresholds usually has three methods: denoising by default threshold, denoising with specified threshold, and forcing threshold. Among them, denoising with a specified threshold is divided into soft threshold and hard threshold. In this paper, a soft threshold method is selected to estimate threshold.

## 3. Denoising Algorithm for Underwater Acoustic Signal

**Figure 4:**
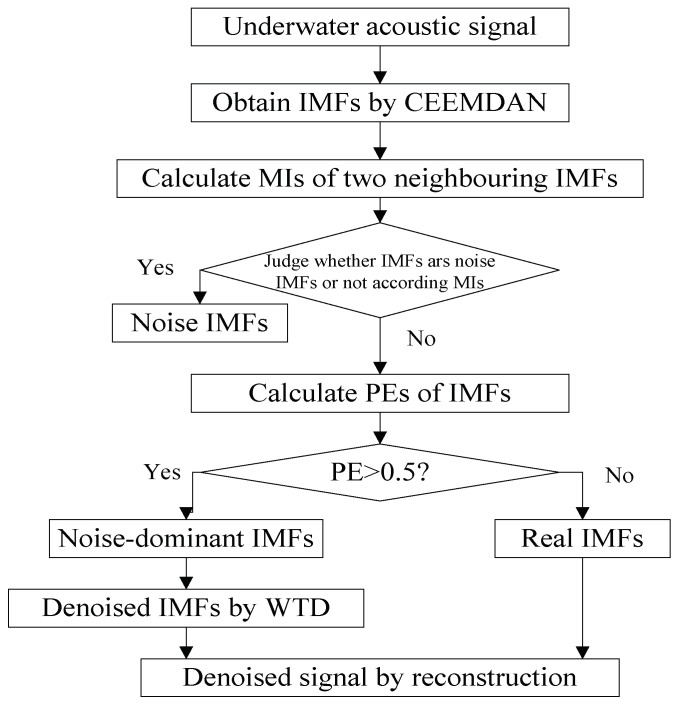
The flow chart of the proposed denoising algorithm for underwater acoustic signal.

The proposed denoising algorithm for underwater acoustic signal is designed in Figure 4. The specific procedures are summarized as follows:

(1) The underwater signal is decomposed by CEEMDAN, we can obtain a lot of IMFs, which contain noise IMFs, noise-dominant IMFs, and real IMFs.
(2) Calculate MIs of two neighboring IMFs in ascending order.
(3) Identify noise IMF according to MIs. If the MI of the K-th IMF and (K+1)-th IMF increases obviously than the former MIs, the former K−1 IMFs are considered as noise IMFs.
(4) Screen out noise IMFs and calculate the PEs of the other IMFs.
(5) Identify noise-dominant IMF according to PEs. If the PE of IMF is more than 0.5, weconsider it as noise-dominant IMF, otherwise real IMF.
(6) Denoise noise-dominant IMFs by wavelet threshold denoising (WTD). We use the wavelet soft threshold denoising for noise-dominant IMFs, wavelet basis function, and decomposition level are db4 and 4, respectively.
(7) The denoised signal can be obtain by reconstructing denoised noise-dominant IMFs and real IMFs.

## 4. Denoising for Simulation Signal

### 4.1. CEEMDAN for Simulation Signal

**Figure 5:**
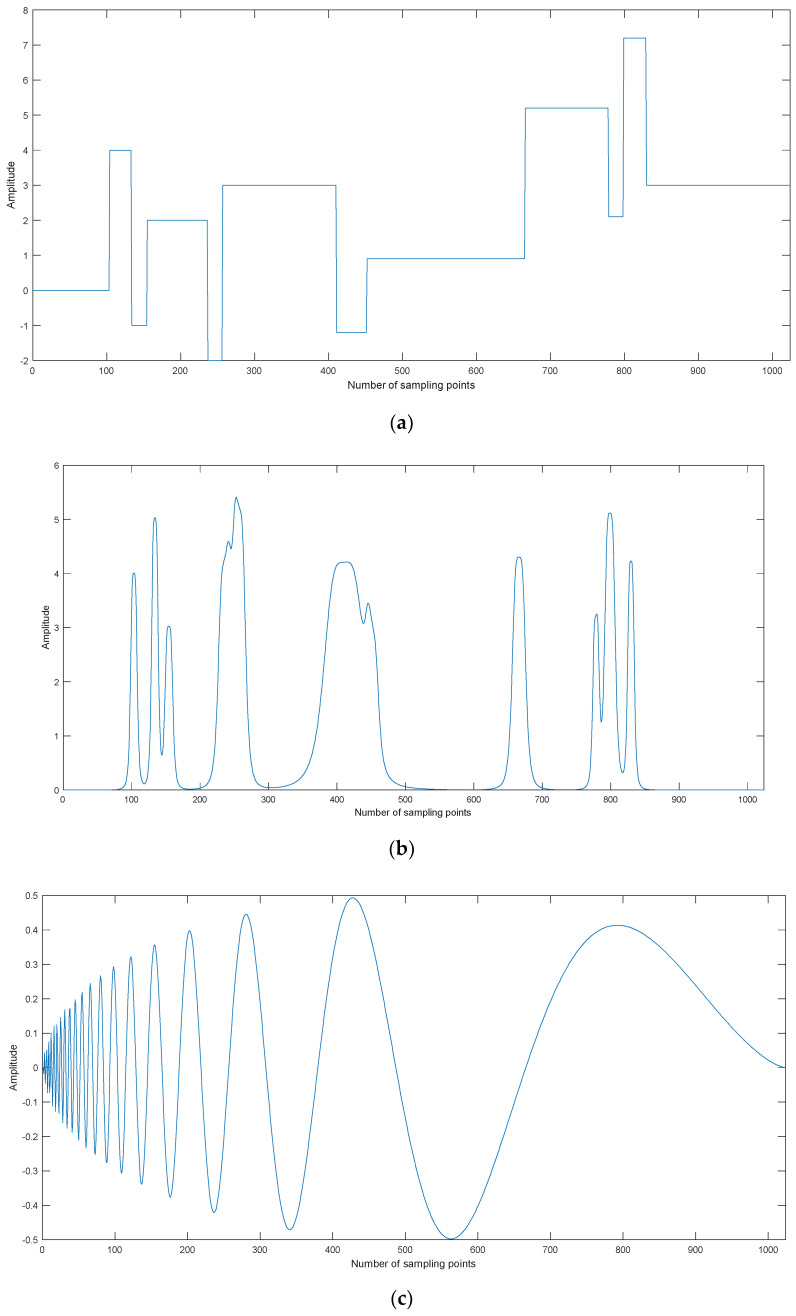

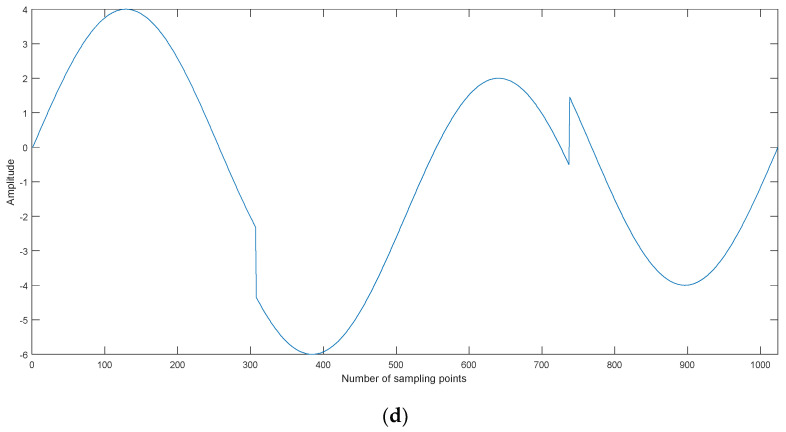
The time-domain waveforms for simulation signals. (**a**) Blocks, **(b**) Bumps, (**c**) Doppler, and (**d**) Heavysine.

Four kinds of simulation signals are selected for denoising, namely, Blocks, Bumps, Doppler, and Heavysine, as shown in Figure 5. The sampling frequency and data lengthare 1 kHz and 1024, respectively.

**Figure 6:**
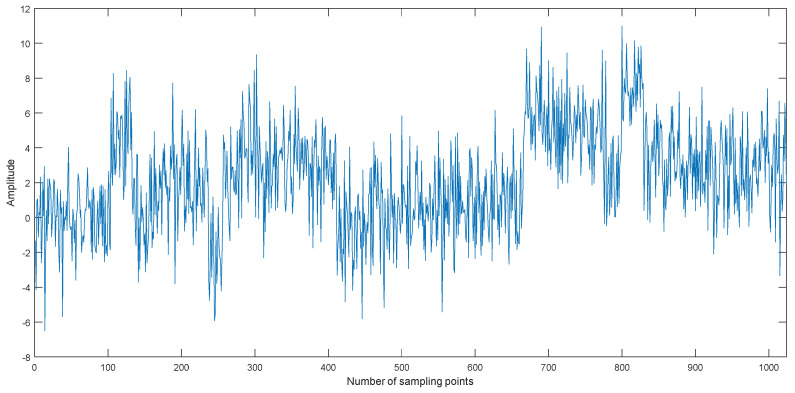
The time-domain waveform of the noisy Blocks signal with 0 dB.

**Figure 7:**
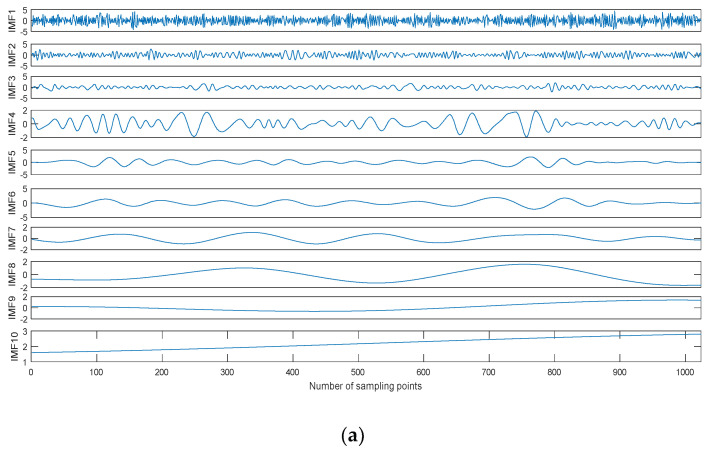

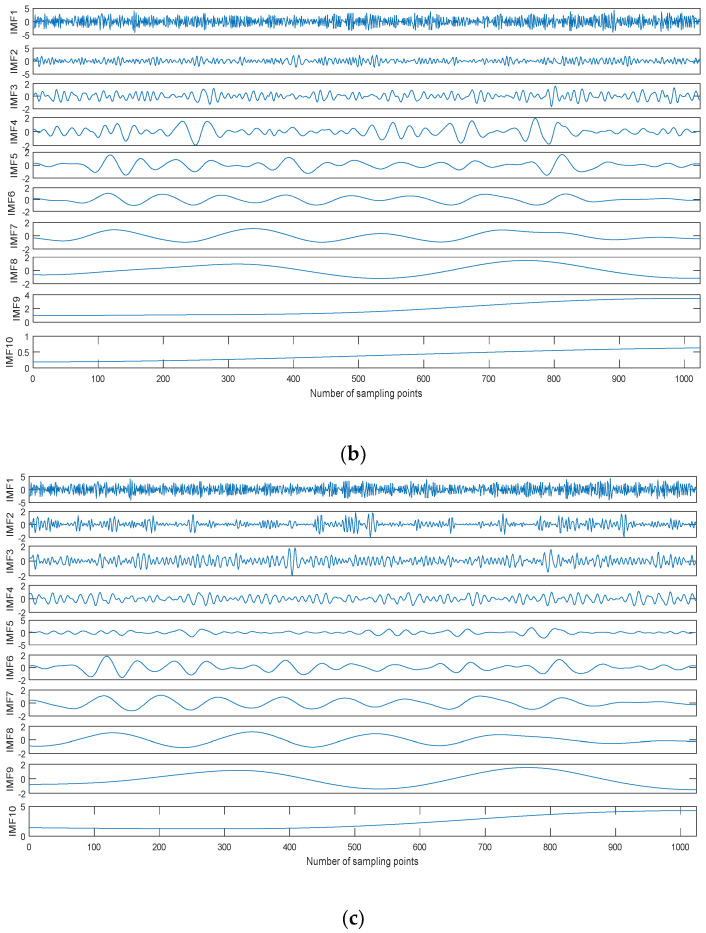
The decomposition result of the noisy Blocks signal with 0 dB. (**a**) Empirical mode decomposition (EMD), (**b**) Ensemble EMD (EEMD), and (**c**) CEEMDAN.

Taking the Blocks signal as an example, we can obtain the noisy Blocks signal with 0 dB signal-to-noise ratio (SNR) by adding Gaussian white noise. The time-domain waveform of the noisy Blocks signal with 0 dB is shown in Figure 6, and the decomposition result is shown in Figure 7. As shown in Figure 6, the Blocks signal has been completely submerged in noise. The noisy Blocks signal is decomposed using EMD, EEMD, and CEEMDAN.As shown in Figure 7, ten IMFs are obtained by three kinds of methods, however, there are some differences for different decomposition methods. IMF1 of each decomposition methods represent the shortest oscillation period, typically a noise component or the high frequency components.

### 4.2. Identifying Noise IMFs

**Figure 8:**
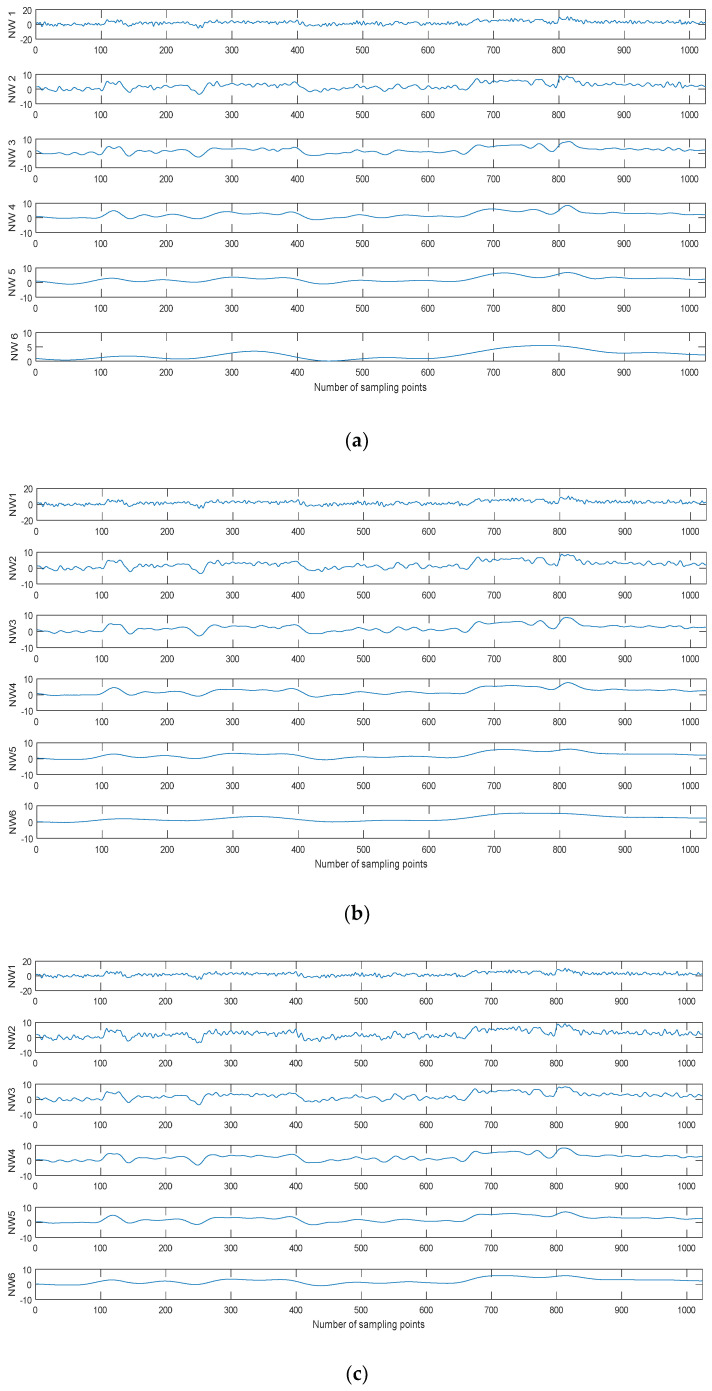
The six kinds of reconstructed signals by different decomposition methods. (**a**) EMD, (**b**) EEMD, and (**c**) CEEMDAN.

In order to observe the effect of noise IMFs on denoising effect, we define the NWn signal as follows:

(25) $$NWn=x(t)-\sum_{i=1}^{n}IMFi(n=1,2,\cdots ,N)$$

where x(t) and N represent the noisy signal and the number of IMF by CEEMDAN, NWn is the restructured signal by removing the first N IMFs. For the noisy Blocks signal with 0 dB SNR, the six kinds of reconstructed signals are shown in Figure 8 using different decomposition methods.As shown in Figure 8, the noise IMFs is eliminated and the reconstructed signal becomes more smoothwith the increasing of n. When n is larger than a certain value, the non-noise IMF is eliminatedand the reconstructed signal is obviously different from the original signal. Therefore, how to identify noise IMFs is the key problem for denoising.

MIs of two neighboring IMFs can expressed as:

(26) $$M_{n}=\mathrm{MI}({\mathrm{IMF}}_{n},{\mathrm{IMF}}_{n+1})(n=1,2,\cdots ,N-1)$$

where $M_{n}$ represents the MI of ${\mathrm{IMF}}_{n}$ and ${\mathrm{IMF}}_{n+1}$. Usually, MI of two noise IMFs is obviously less than the MI of two non-noise IMFs. Therefore, when $M_{n}$ increases obviously, the former n−1 IMFs can be judged as noise IMFs.

For the noisy Blocks signal with 0 dB SNR, MIs of two neighboring IMFs by different decomposition methods are shown in Table 3. As shown in Table 3, $M_{4}$ is more than the former ones for EMD and EEMD, we can judge the first three IMFs as noise IMFs. Similarly, the first four IMFs are noise IMFs for CEEMDAN.

### 4.3. Identifying Noise-Dominant IMFs

Noise-dominant IMFs can be identified according to PEs of non-noise IMFs. For the noisy Blocks signal with 0 dB SNR, PEs of non-noise IMFs are shown in Table 4. As shown in Table 4, the PE of IMF5 is more than 0.5, IMF5 is the noise-dominant IMF for CEEMDAN; real IMFs are the last five IMFs.

### 4.4. Denoising for Noise-Dominant IMFs and Reconstruction

**Figure 9:**
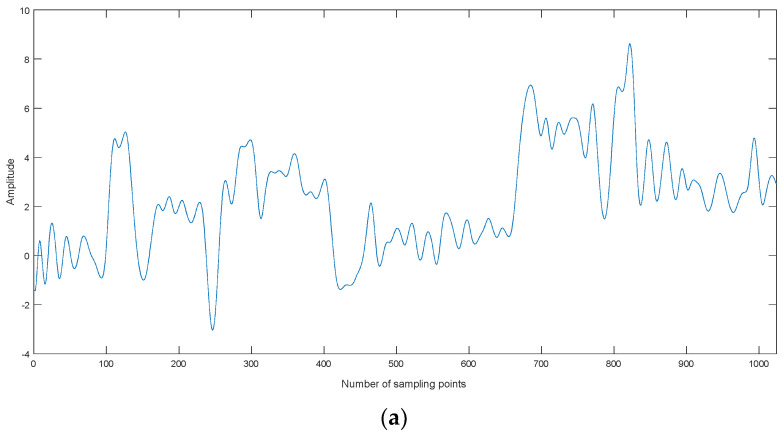

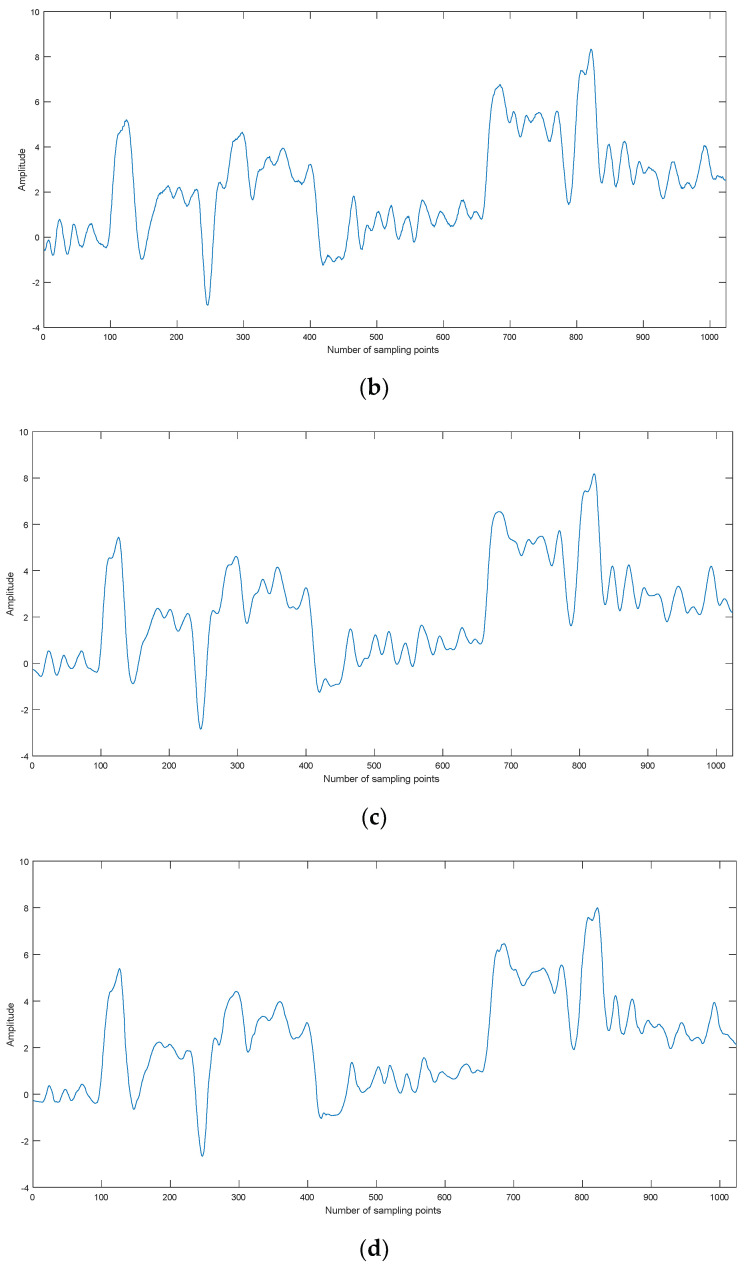
The denoising results for different methods. (**a**) EMD-MI, (**b**) EEMD-MI, (**c**) CEEMDAN-MI, and (**d**) CEEMDAN-MI-PE.

The wavelet soft-threshold denoising is applied to IMF5, the wavelet basis function and decomposition level are db4 and 4, respectively. The denoised Blocks signal is obtainedby reconstructing denoised IMF5 and real IMFs. The denoising results are shown in Figure 9. Denoising methods using MI combined with EMD, EEMD, and CEEMDAN are called EMD-MI, EEMD-MI, and CEEMDAN-MI, theproposed denoising method is calledCEEMDAN-MI-PE.

The parameters of different denoising methods are shown in Table 5. As shown in Table 5, theproposed denoising method has lower root mean square error (RMSE) and higher SNR, which outperforms other three denoising methods.

### 4.5. Comparison of Different Denoising Methods

#### 4.5.1. Wavelet Denoising

The wavelet soft-threshold denoising (WSTD) is applied to four kinds of noisy signals with different SNR, wavelet basis function is db4, decomposition level is from 1 to 6. WSTD results are shown in Table 6. As shown in Table 6, SNRs of the four kinds of signals increase with the increasing of decomposition levels. When the decomposition level increases to a certain value, the SNR reaches a maximum. For Doppler and Heavysine signals, when the decomposition level is 5, the denoising results are optimal. For Blocks and Bumps signals with different SNRs, the optimal denoising effects are distributed in different decomposition levels.

#### 4.5.2. Comparison of Denoising Effect

Four kinds of signals with different SNRs are denoised by EMD-MI, EEMD-MI, CEEMDAN-MI, CEEMDAN-MI-PE, and WSTD. Denoising results of different methods are shown in Table 7, where WSTD denoising results are optimal values in Table 6. All the results of SNRs and RMSEs are the mean of 500 simulations. As shown in Table 7, the CEEMDAN-MI is better than EMD-MI, EEMD-MI, and WSTD, the CEEMDAN-MI-PE has lower RMSE and higher SNR, which has a better performance than the other four denoising methods.

## 5. Denoising for Chaotic Signal

Underwater acoustic signals have the chaotic characteristic, a typical Lorenz chaotic system is used to test the effectiveness of the CEEMDAN-MI-PE denoising algorithm.

The Lorenz system can be expressed as:

(27) $$\left[\begin{array}{l} \dot{x}\\ \dot{y}\\ \dot{z} \end{array}\right]=\left[\begin{array}{ccc} -A & A & 0\\ C & -1 & 0\\ 0 & 0 & -B \end{array}\right]\left[\begin{array}{c} x\\ y\\ z \end{array}\right]+\left[\begin{array}{c} 0\\ -xz\\ xy \end{array}\right]$$

where A is 10, B is 8/3, C is 28.

The Runge–Kutta iteration method is used to calculate the x component with a step length of 0.01.The *x* component signal with a length of 2000 points is selected as Lorenz signal, and the Lorenz noisy signal with different SNR are obtained for CEEMDAN-MI-PE denoising.

**Figure 10:**
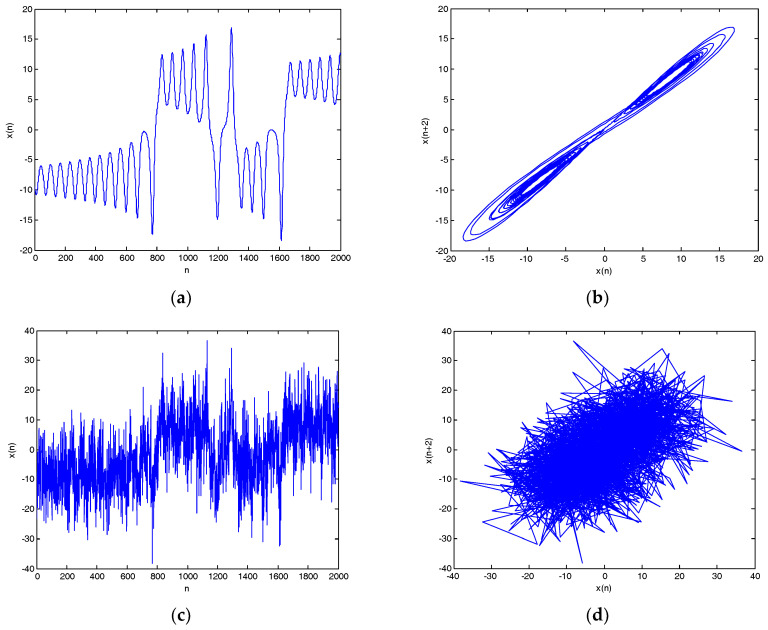

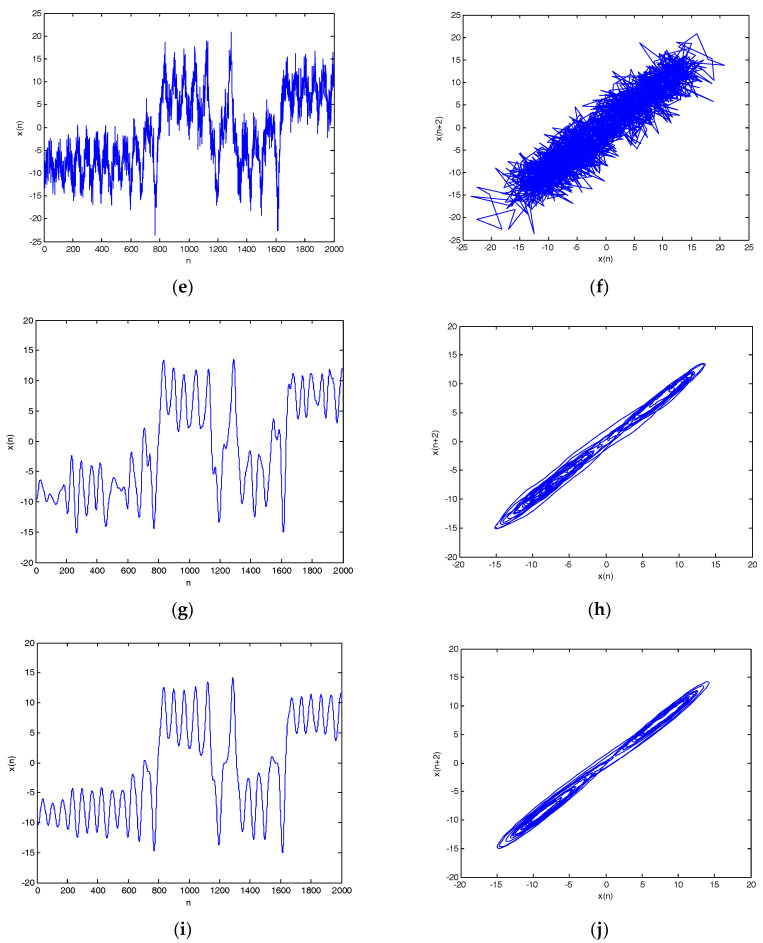
Lorenz noisy and denoised signals with different SNRs and their attractor trajectories. (**a**) Lorenz signal, (**b**) Lorenz attractor trajectory, (**c**) Lorenz noisy signal with 0 dB, (**d**) Noisy attractor trajectory with 0 dB, (**e**) Lorenz noisy signal with 10 dB, (**f**) noisy attractor trajectory with 10 dB, (**g**) denoised Lorenz signal with 0 dB, (**h**) denoised attractor trajectory (0 dB), (**i**) denoised Lorenz signal with 10 dB, and(**j**) denoised attractor trajectory (10 dB).

Lorenz noisy and denoised signals with different SNRs and their chaotic attractor trajectories are shown in Figure 10. As shown in Figure 10, denoised Lorenz signals and their chaotic attractor trajectories by CEEMDAN-MI-PE are close to Lorenz signal and its attractor trajectory, the denoised chaotic attractor trajectories are more smooth and regular.

Denoising results of different SNR by CEEMDAN-MI-PE are shown in Table 8. As shown in Table 8, the SNR and RMSE are improved evidently, the proposed denoising method enhances the SNR more than 10 dB. Overall, the above results show that the CEEMDAN-MI-PE method is suitable for chaotic signals.

## 6. Denoising for Underwater Acoustic Signal

**Figure 11:**
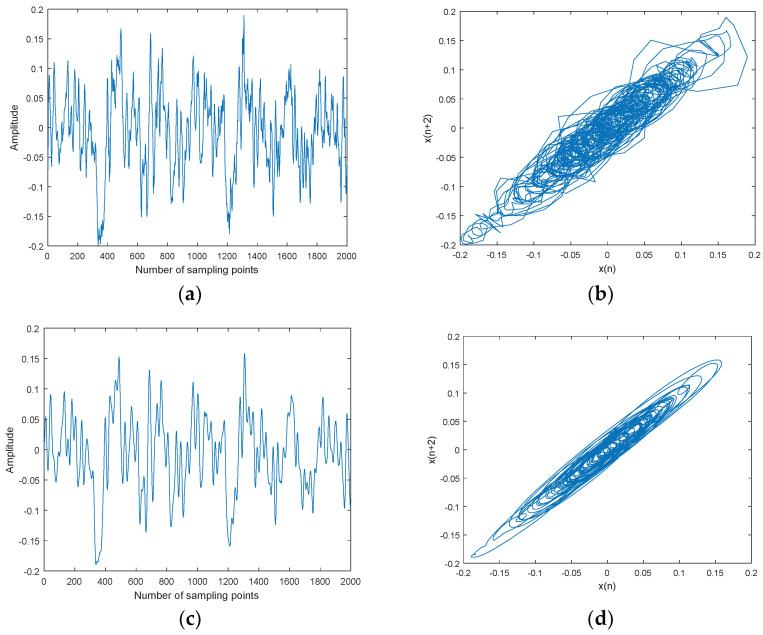
Ship-1 and denoised Ship-1 signals and their attractor trajectories. (**a**) Ship-1, (**b**) attractor trajectory for ship-1, (**c**) denoised Ship-1, and (**d**) attractor trajectory for denoised ship-1.

**Figure 12:**
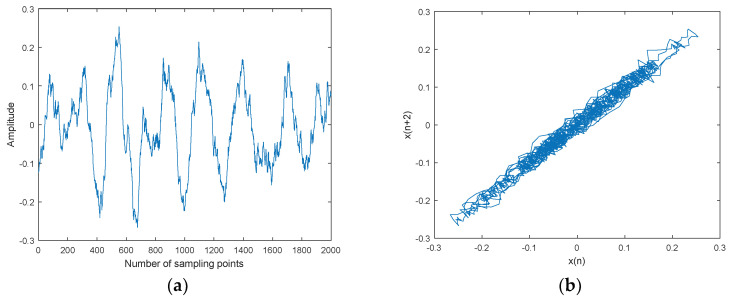

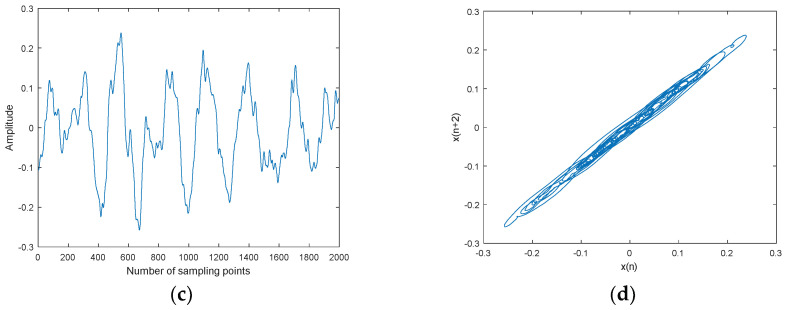
Ship-2 and denoised Ship-2 signals and their attractor trajectories. (**a**) Ship-2, (**b**) attractor trajectory for ship-2, (**c**) denoised Ship-2, (**d**) attractor trajectory for denoised ship-2.

**Figure 13:**
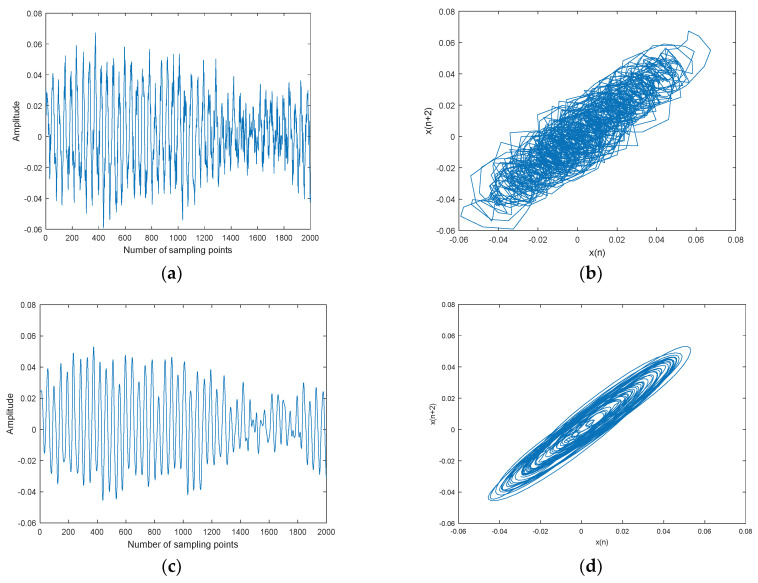
Ship-3 and denoised Ship-3 signals and their attractor trajectories. (**a**) Ship-3, (**b**) attractor trajectory for ship-3, (**c**) denoised Ship-3, (**d**) attractor trajectory for denoised ship-3.

The CEEMDAN-MI-PE denoising is applied to three kinds of underwater acoustic signals, namely ship-1, ship-2, and ship-3. Three kinds of ship signals were recorded by calibratedomnidirectional hydrophones at a depth of 29 m in the South China Sea. During recording, there wereno observed disturbances from biological or man-made sources. The distance between the ship andhydrophone was about 1 km. The sampling frequency was set as 44.1 kHz. Ship signals and denoised ship signals and their attractor trajectories are shown in Figure 11, Figure 12 and Figure 13. As shown in Figure 11, Figure 12 and Figure 13, ocean background noiseis included in original ship signal, high frequency noise is removed effectively by CEEMDAN-MI-PE, denoised attractor trajectories of ship signals are more regular than original ones.

Denoising results of different ships by CEEMDAN-MI-PE are shown in Table 9. Two kinds of PE were used to evaluate the effect of denoising. PE can represent the complexity of time series. A new PE (NPE) was proposed in a previous paper [32], and is interpreted as the distance to noise, which shows a reverse trend to PE. As shown in Table 9, the PE after denoising is less than the one before denoising, which means that the complexity is reduced by denoising; the NPE after denoising is more than the one before denoising, which means that the distance to noise is increased by denoising. In summary, the above results show that the CEEMDAN-MI-PE method is effective and suitable for underwater acoustic signals.

## 7. Conclusions

To improve the denoising effect of underwater acoustic signal, a new denoising method is proposed based on CEEMDAN, MI, PE, and WSTD. CEEMDAN is used to decompose noisy signal into IMFs, noise IMFs, and noise-dominant IMFs which can be identified by MI and PE, WSTD is used for denoising noise-dominant IMFs. The innovations and conclusions of the proposed denoising method are as follows:

(1) CEEMDAN, as an adaptive decomposition algorithm, is introduced for underwater acoustic signal denoising.
(2) Compared with existing denoising methods, IMFs by CEEMDAN are divided into three parts (noise IMFs, noise-dominant IMFs, and real IMFs) for the first time.
(3) Four kinds of signals (Blocks, Bumps, Doppler, and Heavysine) with different SNRs are denoised by EMD-MI, EEMD-MI, CEEMDAN-MI, CEEMDAN-MI-PE, and WSTD, the proposed denoising method has lower RMSE and higher SNR, which has a better performance.
(4) For chaotic signals with different SNR and underwater acoustic signals, the CEEMDAN-MI-PE is also an effective denoising method, which is beneficial to the subsequent processing of underwater acoustic signals.

## Figures and Tables

**Table 1:** The center frequency distribution of intrinsic mode functions (IMFs).

IMF1	IMF2	IMF3	IMF4	IMF5	IMF6	IMF7	IMF8	IMF9	IMF10
12333	9068.1	6296.3	3065.5	1595.4	902.08	446.55	340.21	127.43	67.039

**Table 2:** The mutual informations (MIs) of two neighboring IMFs.

MI_1_	MI_2_	MI_3_	MI_4_	MI_5_	MI_6_	MI_7_	MI_8_	MI_9_
0.0676	0.0501	0.0511	0.1279	0.1751	0.3437	0.8394	0.9998	1.6231

**Table 3:** MIs of two neighboring IMFs by different decomposition methods.

Methods	$M_{1}$	$M_{2}$	$M_{3}$	$M_{4}$	$M_{5}$	$M_{6}$	$M_{7}$	$M_{8}$	$M_{9}$
EMD	0.0034	0.0113	0.0633	0.1805	0.4233	0.6419	1.332	2.1514	3.0829
EEMD	0.0014	0.0023	0.0475	0.1969	0.5045	0.8873	1.8319	1.5387	3.0034
CEEMDAN	0.0143	0.0419	0.0647	0.0803	0.2169	0.5376	0.8044	1.5476	2.3663

**Table 4:** PEs of non-noise IMFs.

IMF5	IMF6	IMF7	IMF8	IMF9	IMF10
0.5869	0.4945	0.4547	0.4263	0.4038	0.3767

**Table 5:** Comparison of the parameters of different denoising methods.

Parameter	EMD-MI	EEMD-MI	CEEMDAN-MI	CEEMDAN-MI-PE
SNR/dB	7.1052	8.6122	9.0433	9.3663
RMSE	0.8031	0.7496	0.7189	0.7078

**Table 6:** (**a**) wavelet soft-threshold denoising (WSTD) results for Blocks signal. (**b**) WSTD results for Bumps signal. (**c**) WSTD results for Doppler signal. (**d**) WSTD results for Heavysine signal.

SNR	Parameter	Decomposition Level					
		1	2	3	4	5	6
(**a**)							
−10 dB	SNR/db	−7.2271	−4.8104	−2.3898	0.2149	1.6290	1.0946
	RMSE	3.6839	3.5798	3.5307	3.5614	3.3392	3.4362
−5 dB	SNR/db	−2.3435	0.3025	3.1728	5.7484	6.3767	3.8336
	RMSE	1.4457	1.5207	1.4748	1.6308	1.5565	1.2164
0 dB	SNR/db	3.2235	6.2477	8.1067	8.4866	7.7998	5.8663
	RMSE	0.8504	0.7895	0.8176	0.8341	0.7473	0.6417
5 dB	SNR/db	7.6526	10.1584	11.4382	10.2663	9.6887	8.4297
	RMSE	0.3915	0.4133	0.4045	0.4114	0.4347	0.4945
(**b**)							
−10 dB	SNR/db	−7.4756	−4.5317	−2.0528	0.3106	0.8154	0.5481
	RMSE	1.6983	1.6584	1.7329	1.6642	1.3528	1.5681
−5 dB	SNR/db	−3.2325	−0.1944	2.3835	4.5283	4.3014	3.5033
	RMSE	1.5826	1.5639	1.5729	1.4888	1.5554	1.9642
0 dB	SNR/db	2.6556	5.1528	7.8573	8.3710	6.9682	6.3942
	RMSE	0.3337	0.3153	0.3046	0.2942	0.3022	0.3025
5 dB	SNR/db	7.4436	10.3617	11.2783	10.7887	9.1181	9.2302
	RMSE	0.2211	0.2385	0.2360	0.2486	0.2182	0.2427
(**c**)							
−10 dB	SNR/db	−6.6660	−4.3199	−0.7130	1.4574	3.4092	3.2951
	RMSE	0.9655	0.9220	0.9301	0.8570	0.9781	1.2149
−5 dB	SNR/db	−1.8736	0.9657	4.1477	6.8774	7.0568	6.4521
	RMSE	0.0502	0.0538	0.0511	0.0491	0.0342	0.0254
0 dB	SNR/db	2.4724	4.5147	8.0985	8.8992	9.1371	8.8475
	RMSE	0.0694	0.0756	0.0545	0.0445	0.0272	0.0284
5 dB	SNR/db	8.1516	10.7101	11.0123	11.2306	11.5458	10.0998
	RMSE	0.0324	0.0333	0.0338	0.0343	0.0285	0.0127
(**d**)							
−10 dB	SNR/db	−6.1624	−3.3838	−0.9295	1.7670	5.8097	4.1349
	RMSE	2.8465	2.6462	2.5926	2.4760	2.6292	4.2984
−5 dB	SNR/db	−2.3572	0.2426	3.1175	6.0666	7.3376	6.857
	RMSE	0.8090	0.8554	0.8105	0.7434	0.7203	0.7268
0 dB	SNR/db	2.9317	5.4369	8.9585	11.8276	14.4169	13.8013
	RMSE	0.2792	0.1840	0.1973	0.1924	0.1682	0.3920
5 dB	SNR/db	8.1963	10.8592	13.4744	15.5710	17.9746	17.6310
	RMSE	0.2759	0.2346	0.2099	0.2277	0.1028	0.1503

**Table 7:** (**a**) Denoising results of different methods for Blocks signal. (**b**) Denoising results of different methods for Bumps signal. (**c**) Denoising results of different methods for Doppler signal. (**d**) Denoising results of different methods for Heavysine signal.

SNR	Parameter	Denoising Method				
		EMD-MI	EEMD-MI	CEEMDAN-MI	CEEMDAN-MI-PE	WSTD
(**a**)						
−10 dB	SNR/db	1.8632	2.0988	2.2803	2.5588	1.6290
	RMSE	5.0621	3.3228	2.5753	2.4237	3.3392
−5 dB	SNR/db	4.8004	6.4972	6.6097	6.8239	6.3767
	RMSE	1.6806	1.4843	1.3438	1.3401	1.5565
0 dB	SNR/db	6.2426	8.4579	9.2502	9.8326	7.7998
	RMSE	0.8261	0.7834	0.7651	0.7051	0.7473
5 dB	SNR/db	11.3699	11.5903	11.7158	11.8733	11.4382
	RMSE	0.6600	0.3917	0.4086	0.3489	0.4045
(**b**)						
−10 dB	SNR/db	−0.1258	0.4728	0.9903	1.2130	0.8154
	RMSE	1.7652	1.4865	1.0045	1.0041	1.3528
−5 dB	SNR/db	3.4745	4.4220	4.6204	4.7355	4.5283
	RMSE	1.8357	1.5325	1.5554	1.4859	1.5888
0 dB	SNR/db	6.8187	7.7571	8.7208	9.0641	8.3710
	RMSE	0.4253	0.3461	0.1969	0.1950	0.2942
5 dB	SNR/db	9.5890	10.5161	11.4614	11.5623	11.2783
	RMSE	0.3158	0.2058	0.1609	0.1513	0.2360
(**c**)						
−10 dB	SNR/db	3.0361	3.3580	3.5508	4.1789	3.4092
	RMSE	1.2158	0.5124	0.4747	0.4597	0.9781
−5 dB	SNR/db	5.7752	6.3432	7.2250	7.2939	7.0568
	RMSE	0.0604	0.0463	0.0263	0.0213	0.0342
0 dB	SNR/db	8.3418	8.6526	8.8555	9.5866	9.1371
	RMSE	0.0235	0.0190	0.0182	0.0165	0.0272
5 dB	SNR/db	11.2457	11.7545	11.8473	12.1583	11.5458
	RMSE	0.0298	0.0025	0.0015	0.0013	0.0285
(**d**)						
−10 dB	SNR/db	6.0781	6.236	6.4919	6.6696	5.8097
	RMSE	1.8252	1.6397	1.5395	1.4666	2.6292
−5 dB	SNR/db	7.1830	8.1239	8.2463	8.3975	7.3376
	RMSE	0.7325	0.6431	0.6324	0.6216	0.7203
0 dB	SNR/db	14.896	15.128	15.2882	15.4476	14.4169
	RMSE	0.1224	0.1158	0.1142	0.1139	0.1682
5 dB	SNR/db	17.8843	19.5125	19.7096	19.7125	17.9746
	RMSE	0.1052	0.0931	0.0925	0.0921	0.1028

**Table 8:** Denoising results of different signal-to-noise ratio (SNR).

SNR	Parameter	CEEMDAN-MI-PE
0 dB	SNR/db	13.254
	RMSE	1.8762
10 dB	SNR/db	20.146
	RMSE	0.3993

**Table 9:** Denoising results of different ships by CEEMDAN-MI-PE.

Parameter		Ship-1	Ship-2	Ship-3
Before denoising	PE	0.8094	0.9231	0.8856
	NPE	0.1227	0.0495	0.0739
After denoising	PE	0.5537	0.5381	0.5148
	NPE	0.2680	0.2765	0.2861
